# Correction: Orbitofrontal neurons acquire responses to ‘valueless’ Pavlovian cues during unblocking

**DOI:** 10.7554/eLife.04741

**Published:** 2014-09-18

**Authors:** Michael A McDannald, Guillem R Esber, Meredyth A Wegener, Heather M Wied, Tzu-Lan Liu, Thomas A Stalnaker, Joshua L Jones, Jason Trageser, Geoffrey Schoenbaum

McDannald MA, Esber GR, Wegener MA, Wied HM, Liu TL, Stalnaker TA, Jones JL, Trageser J, Schoenbaum G. 2014. Orbitofrontal neurons acquire responses to ‘valueless’ Pavlovian cues during unblocking. *eLife* **3**:e02653. doi: http://dx.doi.org/10.7554/eLife.02653. Published 18 July 2014

The original version of this article stated that outcome features might be the underlying basis of apparent value signals in a number of studies. Here we wish to note that in at least one of these studies (Padoa-Schioppa and Asaad, Nature, 2006), the authors argue categorically that their abstract value correlates cannot be explained as signaling reward size, number, or any other feature or ‘ingredient’ of the rewards, either in isolation or any possible combination. By far, the most powerful argument in favor of this position is located in their supplemental data where they show that the ratio of the slope of the firing to the juice pairs used in each session was correlated with the relative value of these juice pairs across sessions. While this relationship could reflect signaling of diverse features of the two juices, the coding of these features would then need to relate to the changes in relative preferences across sessions. Although not inconceivable (since sensory precepts and processing likely do change with the value of or preference for the predicted outcomes), we agree that these supplemental results do rule out simple feature-based explanations of this particular result. Please see their original research article and particularly their online supplemental material for supporting information.

The original paragraph follows:

‘Yet in all these studies, outcome features could still be the underlying basis of the neural signals. This is because a signal that varies with outcome value might be encoding features that co-vary with value. This is true even if the signal correlates with value across different outcomes, since some features will be common across the limited number of outcomes used in any particular experiment. For example, a common neural code that seems to be similar for two juices may track their sweetness or another dimension such as number or size that increases with value. Further, any neural element (voxel or single unit) may participate in ensembles responding to more than one feature, so it is also possible that a particular element that appears not to distinguish specific features of different outcomes is in fact coding independent features that co-vary with each outcome’s value.’

This has been replaced with the following text:

‘Yet in some cases, outcome features could still be the underlying basis of apparent abstract value signals. This is conceivable even if the signal correlates with value across different outcomes, since some features or feature combinations might vary with value across the limited number of outcomes used in any particular session (but see Padoa-Schioppa and Asaad, 2006, supplemental, and our correction notice). Further, any neural element (voxel or single unit) may participate in ensembles responding to more than one feature, so it is also possible that a particular element that appears not to distinguish specific features of different outcomes is in fact coding independent features that co-vary with each outcome's value.’

Additionally, during the typesetting of the legend for Figure 1F, the ANOVA factor of trial (1–15) was mistakenly converted to a citation of references 1–15. The citations have been removed and replaced with the trial numbers.

The article has now been corrected.

